# Development of an advanced velocity modulator for a portable Mössbauer spectrometer

**DOI:** 10.1038/s41598-023-46846-x

**Published:** 2023-11-13

**Authors:** Joanne Yoon, Jaegi Lee, Sung-Joon Ye, Young-bong Bang

**Affiliations:** 1 Graduate School of Convergence Science and Technology, Seoul National University, Seoul, 08826 Republic of Korea; 2 HANARO Utilization Division, Korea Atomic Energy Research Institute, Daejeon, 34057 Republic of Korea; 3 Department of Transdisciplinary Medicine, Seoul National University Hospital, Seoul, 03122 Republic of Korea; 4 College of Medicine, Seoul National University, Seoul, 03080 Republic of Korea

**Keywords:** Engineering, Physics

## Abstract

Mössbauer spectroscopy is a nuclear spectroscopic technique that measures changes in energy on an atomic scale. In a Mössbauer spectrometer, a velocity modulator oscillates a radioactive source to vary the energy of gamma rays. Conventional velocity modulators use wires primarily as motion guides; however, the tension state of these wires may change over time. Membrane springs are thus used as an alternative to wires; however, they also present certain challenges related to their design, manufacturing, and assembly. Instead of wires or membrane springs, this study used a linear bearing with preloaded compression springs. The advantage of this mechanism is that permanent deformation or changes in spring stiffness minimally occur during spring assembly and operation. The developed velocity modulator is compact and light, making it ideal for portable applications. A digital controller is used to easily modify and customize control parameters and the supporting algorithm, which is not easily achieved with conventional analog controllers. Moreover, by applying a switching amplifier, low-power operation is also achieved. Feedforward control values are calculated by an iterative learning method that is robust to the control of repeated motion. Using finite element method simulations and experiments, the performance of the developed prototype was evaluated. The velocity signal demonstrated linearity with a correlation with a straight line of approximately 0.996 for a triangular velocity profile (satisfactory performance).

## Introduction

Mössbauer spectroscopy is a technique based on the Mössbauer effect, which involves resonant and recoil-free emission and absorption of gamma radiation by atomic nuclei bound in a solid^1^. Using Mössbauer spectroscopy, small changes can be measured in the chemical environment of Mössbauer nuclei (e.g., 57-Fe)^2, 3^. There have been many applications of Mössbauer spectroscopy in analytical chemistry and materials science, such as determining the chemical states and crystal structures of iron compounds in cultural assets without damaging them (a.k.a. nondestructive testing)^4–7^.

**Figure 1:**
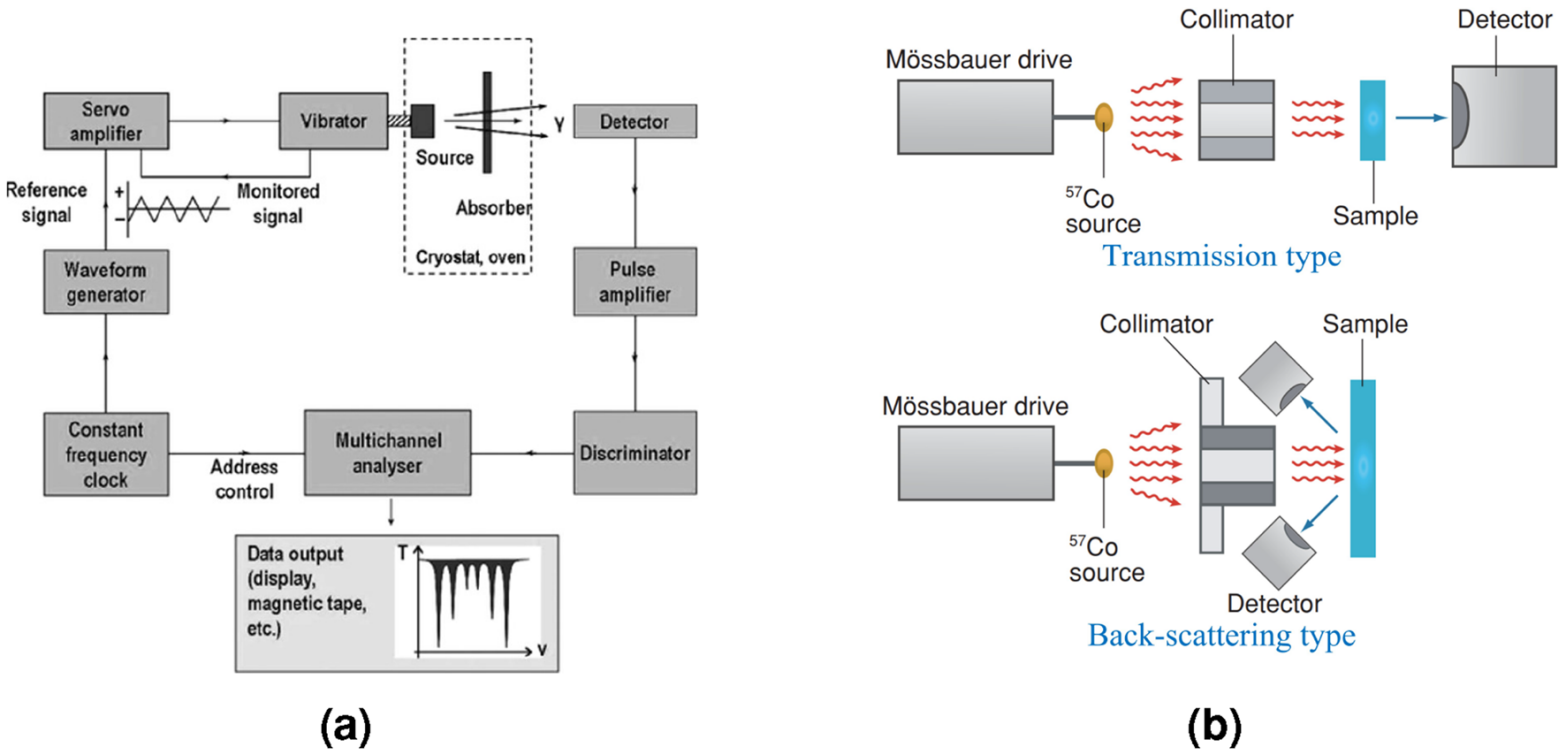
Mössbauer spectrometer. (**a**) Schematic diagram^8^. (**b**) Two types of configurations^6^.

Figure 1a shows the typical configuration of a Mössbauer spectrometer, which comprises three primary components: a radioactive source, a collimator that filters out nonparallel gamma rays, and a photon detector^3, 8^. In Mössbauer spectral measurements, the radioactive source is oscillated by a linear motor in a specific velocity range to generate a Doppler effect. This process allows gamma rays to be shifted by amounts proportional to the velocities of the radioactive source, so gamma ray energy can be scanned over a specific range, and a Mössbauer spectrum can be obtained^3, 9^. A subsystem for driving the gamma ray source is called a velocity modulator, and it generally consists of a linear motor, a linear sensor, and a dedicated servo controller. Since Mössbauer spectroscopy depends on the motion of the gamma ray source, the velocity modulator must be designed for precise and stable motion control.

The basic concepts of the velocity modulator, including a structure with dual coils (driving coil and sensing coil) and its operating principle based on electromagnetic phenomena, were established decades ago^10–12^. The driving coil realizes motion based on the Lorentz force. This actuation unit, also called the voice coil motor (VCM), has a fast response and simple structure. The sensing coil is an analog linear velocity transducer (LVT). It is based on Faraday’s law of induction and outputs a voltage signal proportional to the speed. In applications where precise speed control is required in short strokes, such as velocity modulators, a LVT offers great advantages. Measuring the speed with a digital linear encoder used in a general servo system requires numerical differentiation. In this process, the resolution of the encoder and the limitations of the sampling period may reduce the accuracy of the speed measurements. For the abovementioned reasons, the VCM and LVT are suitable mechanisms for velocity modulators. Therefore, many related studies^10–15^ and commercial products^16–18^ have adopted similar structures. This study investigates the VCM and LVT as the basic components of the velocity modulator.

In conventional velocity modulators, wires or strings (Kapton wires nylon wires, etc.) are commonly used as motion guide springs^11^. Wire-based methods are easy to install, ighten the moving part, and greatly reduce the friction it experiences. Despite the effectiveness of such methods, the tension of the wires or strings may change over time, which reduces the ability to keep the performance of the velocity modulator stable. To address this issue, membrane springs (or thin-plate springs) have been introduced as alternatives to wires or strings^10, 13–15^. Even though membrane springs can maintain tension for a longer period than the wire-based method, it is not simple to design its geometry or pattern so that it has the required stiffness. It is also difficult to precisely machine the designed membrane pattern. Moreover, special care must be taken when assembling these systems. In the early stage of this study, thin-plate springs were considered. However, when springs were installed, they were too prone to torsion, buckling, or permanent deformation. This limit causes nonlinear characteristics that adversely affect speed control. Consequently, this study investigated a linear bearing as a motion guiding part and preloaded compression springs as position returning springs, a method that is clearly different from previous mechanisms (wires or membrane springs).

On the other hand, most studies consider the development of velocity modulators have addressed improvement of the controller as the main topic rather than the mechanical structure. In early velocity modulators, analog controllers using operational amplifiers (op-amps) were usually applied^10–12^. Analog controllers provide high-resolution and fast signal processing speed, but they are vulnerable to noise and require changes in circuit design to modify control parameters and algorithms. Therefore, more recent studies have used a digital controller with a high-speed processor^13, 15, 19–22^. The digital controller can modify and customize control algorithms through software programming. For example, an algorithm was reported that automatically tuned the values of the control parameters based on a digital controller^21, 22^. However, linear amplifiers were still used as the motor driving circuit^20, 21^. In contrast, a switching amplifier was used in this study. A switching amplifier has better energy efficiency than linear types, and it generates less heat, so it is advantageous for long-term use.

**Table 1:** Performances of commercial velocity modulators^16–18^.

Product	Size (mm$^2$) and weight (kg)	Max. speed (mm/s)	Resonance frequency (Hz)	Velocity sensitivity (mV per 1 mm/s)
MA-250	$\Phi 108\times 178$, 5.2	200	25	30
MA-260S	$\Phi 108\times 200$, 3.9	300	25	25
MVT-1000	$\Phi 108\times 138$, 4.5	300	25	25

This study presents a high-performance velocity modulator for a portable Mössbauer spectrometer. A portable Mössbauer spectrometer is required to handle samples that cannot be brought into the laboratory environment or cultural objects that must not be damaged. It should be a back-scattering type, where a velocity modulator and a gamma-ray detector are integrated into a single housing (Fig. 1b). Therefore, the smaller and lighter the velocity modulator is, the greater the mobility of the Mössbauer spectrometer. Most commercially available velocity modulators are large and heavy (Table 1) because they are made for general purposes that can drive heavy samples at high speed and mainly target transmission configuration. Therefore, a task described herein is the design of a velocity modulator that is more compact and lighter than those used in existing commercial products. For this study, a prototype velocity modulator was developed, which has good performance and distinguishable features from existing devices.

## Methods

### Design requirements

The design requirements for this study were based on the typical operating conditions of a Mössbauer spectrometer targeting 57-Fe samples. A driving velocity range of approximately ±10 mm/s can cover the appropriate energy shifting range of the gamma ray for 57-Fe samples^13^. As Table 1 shows, the maximum speed of commercial products is greater than 10 mm/s for general purposes including samples other than 57-Fe and their corresponding sources. This study targeted only 57-Fe, so there is no need to provide a wide range of speeds similar to commercial products.

When a velocity modulator oscillates the radioactive source, a cyclic triangular waveform is typically the target velocity profile^23–25^. This profile offers the advantage of a shorter dead time at the velocity transition point, where the movement is nonlinear^13^. In the case where the frequency is set to 25 Hz and the amplitude is set to 10 mm/s in the triangular velocity profile, the required stroke is calculated as 100 µm, and the acceleration is 1 m/s^2^. The scanning rate, which is the frequency of the velocity profile, is typically set close to the resonance frequency of the velocity modulation system to maximize energy efficiency^26^. If the scanning rate increases, then the required stroke decreases, but the required acceleration increases. Conversely, if the scanning rate decreases, then the required acceleration decreases, but the required stroke increases. Therefore, when designing a mechanical system and the VCM, it is essential to consider a sufficient margin for both the moving stroke and the acceleration performance (i.e., power) of a linear motor.

For the LVT, the higher the voltage output ratio to the velocity, the higher the sensitivity, and thus the higher the signal-to-noise ratio, which is advantageous for precise control. Here, a design goal has been established for the LVT, which aims to achieve higher sensitivity than commercial products (Table 1).

### Mechanical system design

**Figure 2:**
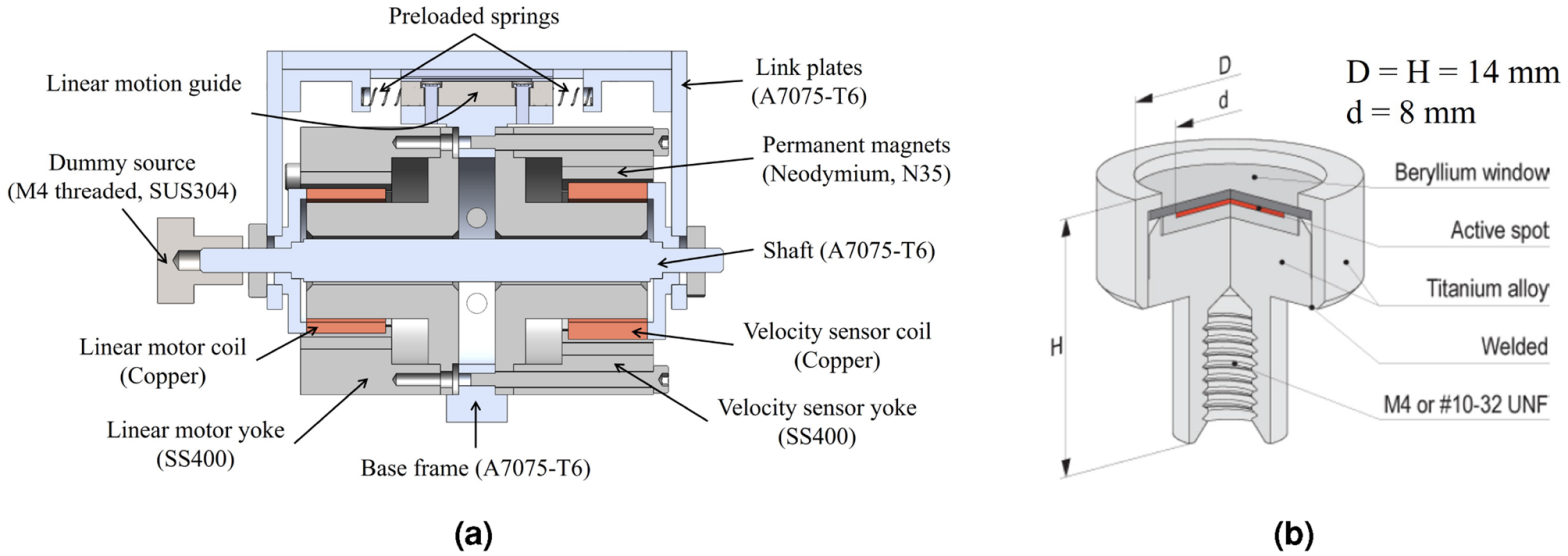
Designed velocity modulator. (**a**) Structure and components. (**b**) Mössbauer source^27^.

Figure 2a illustrates the structure and major components of the proposed velocity modulator. A linear motion guide is essential for reducing friction in the driving direction and for supporting lateral forces. This design used a commercial linear bearing of the ball-slide type (BSGM6-25, Misumi) as a linear motion guide, rather than wires or membrane springs. The selected linear bearing has low friction and stable movement. Although it makes moving parts slightly heavier than those made with wires or membrane springs, it is more mechanically durable.

The mechanical structure can achieve better stability when two linear bearings are employed and symmetrically installed with respect to the moving shaft. However, if the axes of motion of the two guides are not accurately aligned with each other and along the moving axis, the design may result in an increase in frictional resistance to the moving part. Therefore, in this study, one linear bearing is utilized.

To limit the position range of the moving part and to prevent position drift during velocity control, commercial compression springs (WF3-10, Misumi) were used as returning springs. Compared to wire or membrane springs, compression springs offer a longer lifespan, a reduced risk of permanent deformation during assembly, and minimal change in spring stiffness. Furthermore, compression springs are commercially available with a variety of specifications, so they are easy to use and replace.

The compression springs are installed on both sides of the linear bearing, each slightly preloaded to prevent easy disassembling. Due to the restoring force from the preload of the springs, the position of the moving part is maintained at the center without drifting toward either end of the stroke, in the idle state.

The stationary parts of the VCM and LVT are fixed to the base frame, and both components have a hollow structure, through which the moving shaft passes at their centers. The movable parts of the VCM and LVT are securely connected to either end of the shaft, moving as a single rigid body. Additionally, the shaft is connected to the carrier of the linear bearing through link plates, and the rail of the linear bearing is fixed to the base frame.

A radioactive source is installed on the moving shaft via the M4 thread. An example of an available Mössbauer source is shown in Fig. 2b. For this study, a stainless-steel (SUS304) dummy source using the same dimensions and weight as the source in Fig. 2b was constructed and used in the “Experiment” section.

### Electromagnetic system design

**Figure 3:**
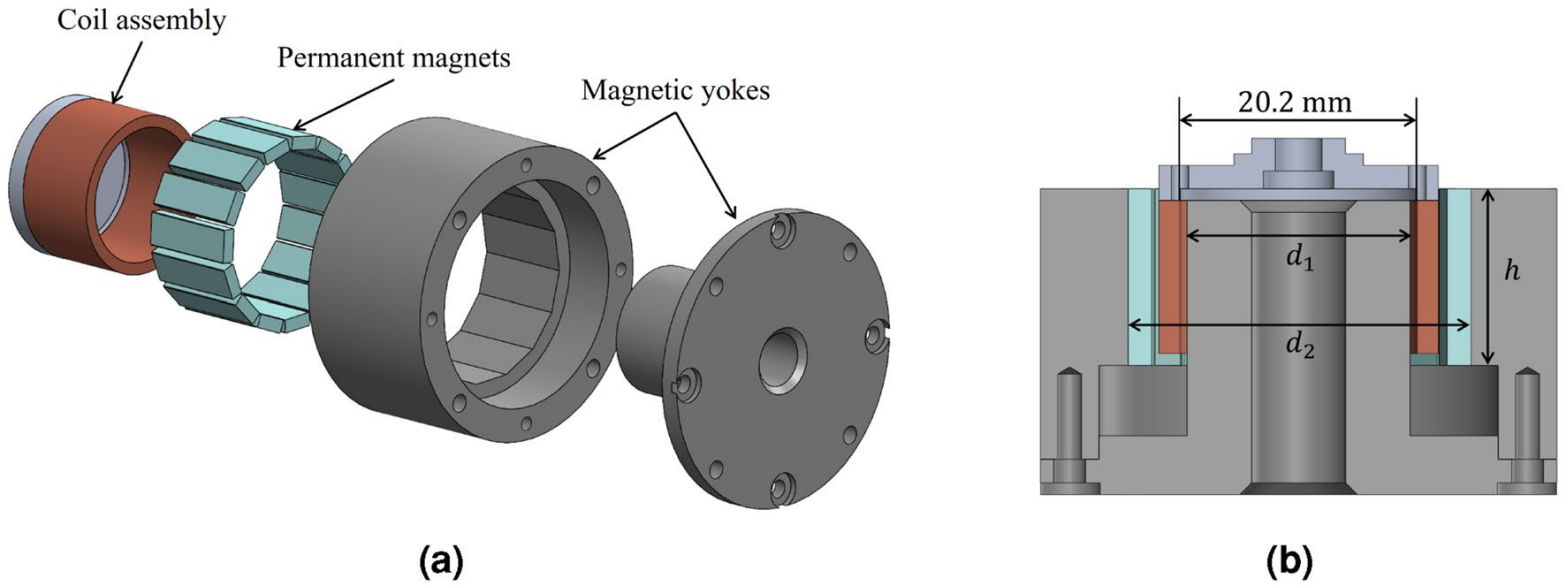
Structure of the VCM and LVT. (**a**) Exploded view. (**b**) Cross section.

The structures of the VCM and LVT are designed in similar geometries, as shown in Fig. 3. Unlike many existing velocity modulators that use axially magnetized permanent magnets in the magnetic core; in this study, radially magnetized magnets are employed to minimize magnetic leakage and reduce energy losses. For this purpose, bar-shaped neodymium permanent magnets (N35, $15\times 5\times 2$T) are attached to the inner surface of each yoke (a low carbon steel, SS400) and arranged in the circumferential direction for magnetic flux (as shown in Fig. 3a) to pass through in a radial direction. Use of a circular array of bar-shaped magnets is a cost-effective and feasible alternative to a toroid magnet with a rectangular section and radial magnetization.

The dimensions $d_1$, $d_2$, and *h* as indicated in Fig. 3b are important parameters in the magnetic circuits of both the VCM and LVT, as they influence the magnetic flux density within the air gap. These parameters are correlated with the number of elements in their magnet arrays, as well as the dimensions and the number of turns in their solenoid coils.

Several constraints existed in the design of the yoke. First, $d_1$ needed to have some dimensional margin compared to the outer diameter of the solenoid coil to prevent interference with the mechanical movement of the solenoid coil. In this study, a coil winding jig with a diameter of 20.2 mm was employed for both the VCM and LVT to reduce coil manufacturing costs. Consequently, the flexibility to adjust $d_1$ was limited. Second, the dimension of $d_2$ had to be determined based on the number of bar-shaped magnets that could be arranged on the yoke in the circumferential direction. Third, the value of *h* had to be based on the height of the magnet.

#### Solenoid coils

The determination of coil turns should be approached distinctly for VCM and LVT systems, depending on their respective requirements as actuators and sensors. In the case of a VCM, increasing the number of coil turns can lead to higher force output, even with a reduced coil current, which effectively increases the force constant (force-to-current ratio). However, as the number of coil turns increases, the coil resistance increases correspondingly, which may necessitate a higher supply voltage to maintain the same current value. Therefore, achieving a comprehensive balance between VCM coil turns, resistance, and supply voltage is crucial. In this study, electromagnetic (EM) static analysis was used to estimate the force constant of the VCM depending on the coil parameters (Flux 2D, Altair).

For an LVT, increasing the number of coil turns within the same volume can lead to an increase in the coil voltage output for the same coil velocity, effectively enhancing the measurement sensitivity. Therefore, a copper wire of thin diameter was selected to increase the number of turns in a limited space. In this study, EM transient analysis was used to estimate the sensitivity of the LVT depending on the coil parameters and coil movement (Flux 2D, Altair).

Table 2 summarizes the design parameters for the VCM and LVT, identifying the appropriate parameter values through trial and error. Considering the size of the yoke and magnet thickness, there was a slight margin in the diameters of the solenoid coils for both the VCM and LVT. The coil heights were adjusted to be less than the height of the permanent magnet, thereby increasing the utilization rate of the magnetic flux in the air gap.

**Table 2:** Design parameters of the EM system.

Parameter	Value	Unit
VCM		
Coil diameter	0.25	mm
Coil turns	305	–
Coil resistance	8	$\Omega$
Coil dimension	$23.8\times 20.2\times 13$	mm$^3$
Yoke dimension ($d_1\times d_2 \times h$)	$19\times 29\times 15$	mm$^3$
LVT		
Coil diameter	0.1	mm
Coil turns	2288	–
Coil resistance	368	$\Omega$
Coil dimension	$25.8\times 20.2\times 13$	mm$^3$
Yoke dimension ($d_1\times d_2 \times h$)	$19\times 31\times 15$	mm$^3$

#### Electromagnetic analysis

In EM analysis of a VCM, it is possible to estimate the permeance line of the magnetic core, determine the operating point of the permanent magnets and calculate the resulting flux density in the air gap. Subsequently, the force and force constant of a VCM can be computed using the formula for the Lorentz force:

1 $$\begin{aligned} { \vec{{F}} = \int i (d \vec{{l}} \times \vec{{B}})} \end{aligned}$$

**Figure 4:**
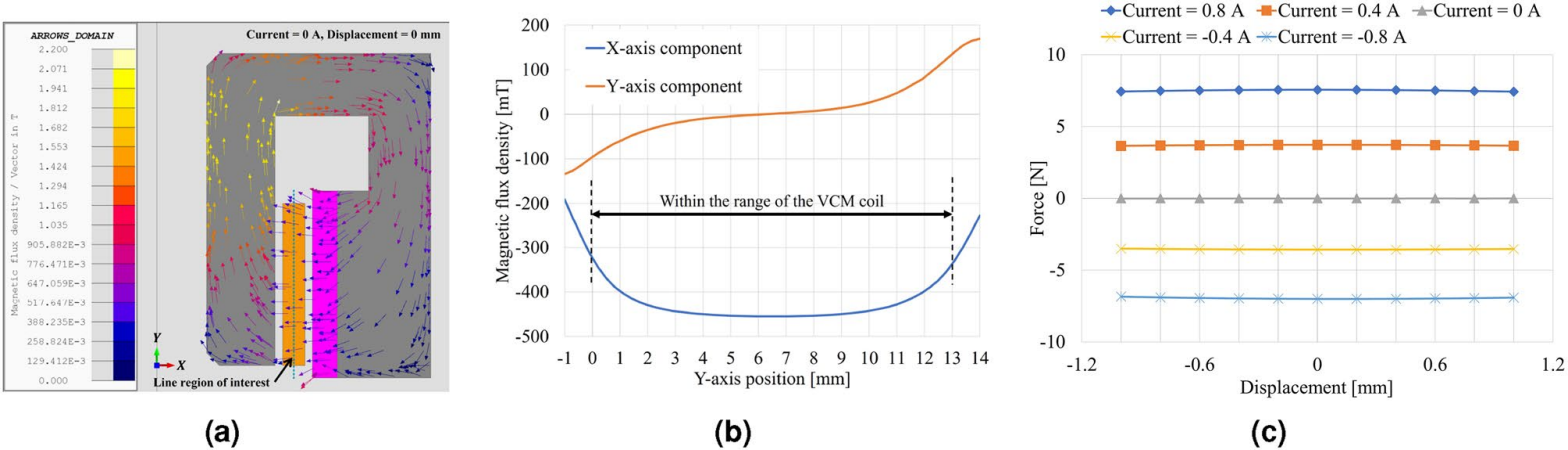
Results of EM analysis of the VCM. (**a**) Magnetic flux density distribution. (**b**) Magnetic flux density in the line region of interest (middle of the air gap). (**c**) Force–displacement property.

The EM analysis for the designed VCM are shown in Fig. 4. At the middle position of the air gap through which the VCM coil passes, the magnetic flux density is distributed as shown in Fig. 4b. The force in the direction of motion (i.e., the *Y*-axis in Fig. 4a) is related to the *X*-axis component of the magnetic flux density. As shown in Fig. 4b, the *X*-axis component of the magnetic flux density is relatively uniform in the range of the VCM coil. The force constant of the VCM is calculated to be approximately 9 N/A (Fig. 4c). Therefore, even when the VCM coil current is 1 A, it can generate an acceleration of 1 m/s$^2$ with a moving mass of up to 9 kg. The moving part consists of various mechanical components, including the coil assemblies, shaft, link plates, carrier of a ball-slide bearing, bolts, and nuts. The total mass of this moving part has been designed to be as small as several tens of grams (at most); thus the designed VCM can handle a substantial range of accelerations.

For a LVT, like a VCM, after the permeance line of the magnetic core is determined, the operating point of the permanent magnets and the flux density in the air gap can be estimated, it becomes possible to calculate the induced electromotive force by coil movement and velocity-to-voltage ratio (measurement sensitivity) for a LVT. This calculation is based on Faraday’s law of electromagnetic induction:

2 $$\begin{aligned} {\varepsilon = \int (\vec{v} \times \vec{B}) \cdot d\vec{l}} \end{aligned}$$

**Figure 5:**
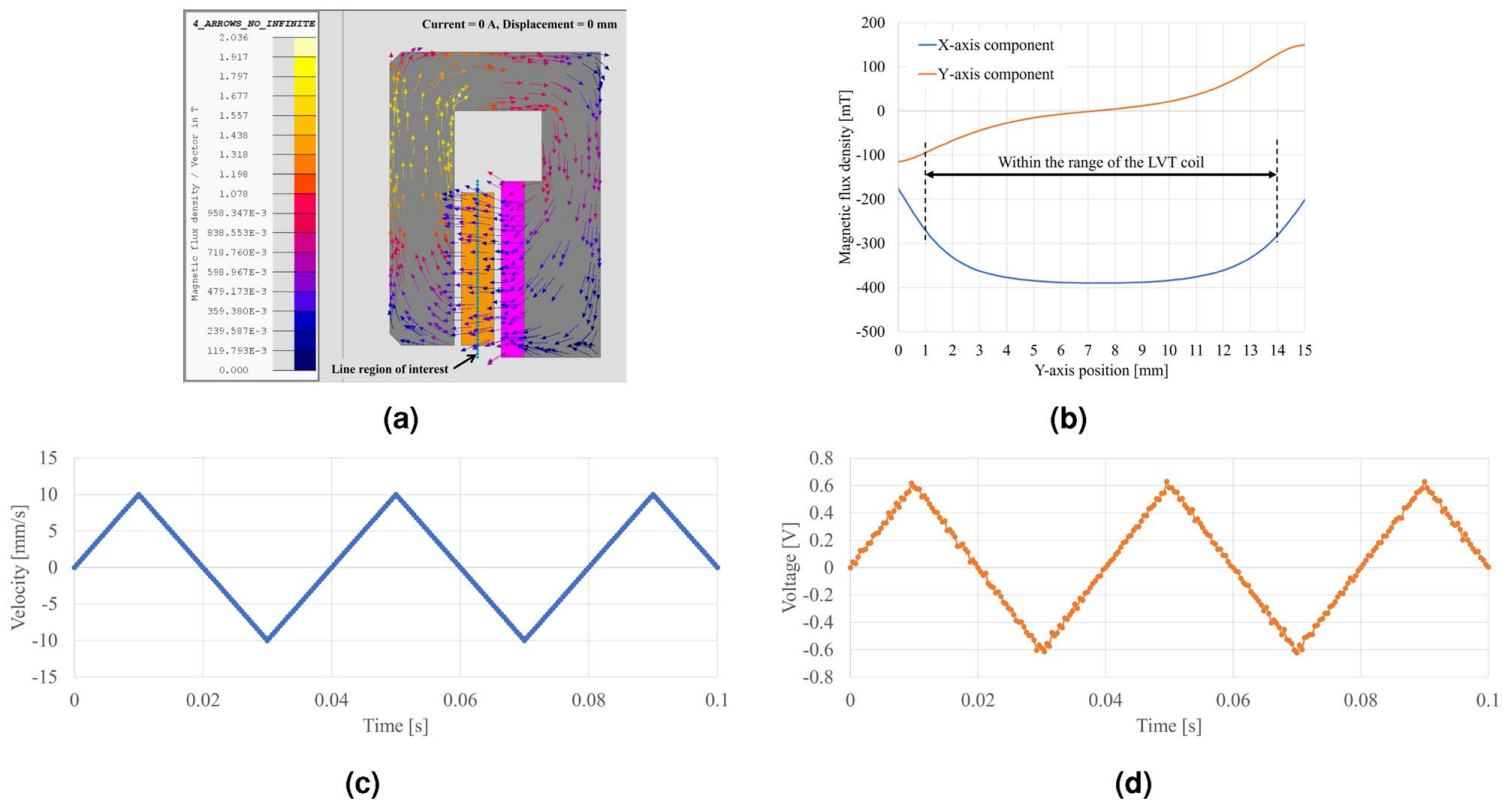
Results of EM analysis of the LVT. (**a**) Magnetic flux density distribution. (**b**) Magnetic flux density in the line region of interest (middle of the air gap). (**c**) Imposed velocity for simulation. (**d**) Simulated LVT signal.

The results of the EM analysis for the designed LVT are presented in Fig. 5. The output voltage of the LVT was simulated while controlling the coil velocity to follow a triangular profile (± 10 mm/s, 25 Hz), with both ends of the coil maintained open (i.e., 0 A). At the middle position of the air gap through which the LVT coil passes, the magnetic flux density is distributed as shown in Fig. 5b. The coil voltage generated for the speed in the moving direction (i.e., the *Y*-axis in Fig. 5a) is related to the *X*-axis component of the magnetic flux density. As shown in Fig. 5b, the *X*-axis component of the magnetic flux density in the range where the coil installed is uniform except for both ends. The sensitivity for 10 mm/s is estimated at aproximately 0.58 V (Fig. 5c,d) (Supplementary Figs. 4 and 5).

**Figure 6:**
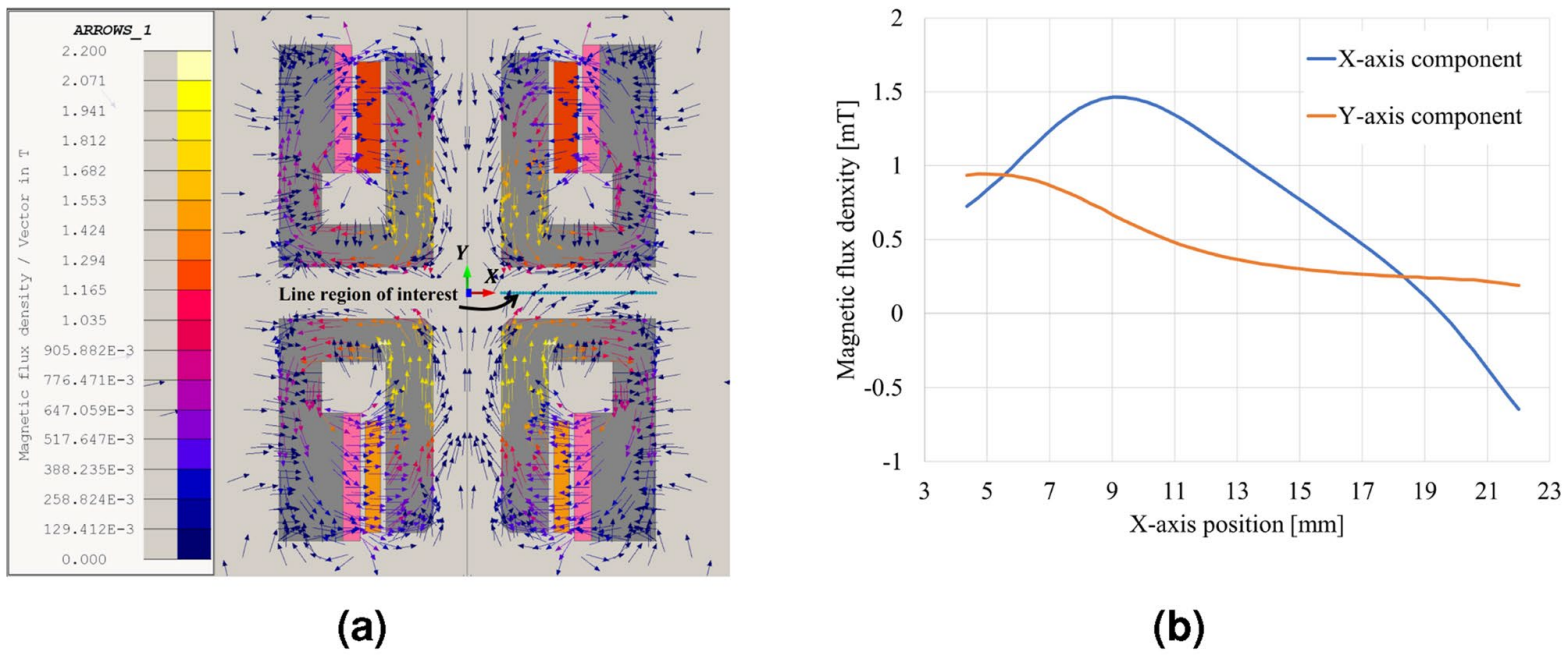
Simulation result of magnetic shielding. (**a**) Magnetic flux density distribution. (**b**) Magnetic flux density in the line region of interest (VCM–LVT middle line).

#### Magnetic shielding

Minimizing magnetic interference between the VCM and LVT is advantageous for reducing noise in the LVT and achieving precise control of the VCM. As depicted in Fig. 2a, the yokes of the VCM and LVT are arranged such that they open in opposite directions. This configuration was determined through EM analysis to evaluate leakage flux and magnetic interference between them. The EM analysis results (Fig. 6) demonstrate that the yokes of the VCM and LVT function as a type of metal barrier, effectively limiting the interference of magnetic flux between these components. The magnetic flux density at the intermediate position between the VCM and LVT is illustrated in Fig. 6b, with the average magnitude of the magnetic flux density measuring less than 2 mT (Supplementary Fig. 6).

### Control system design

**Figure 7:**
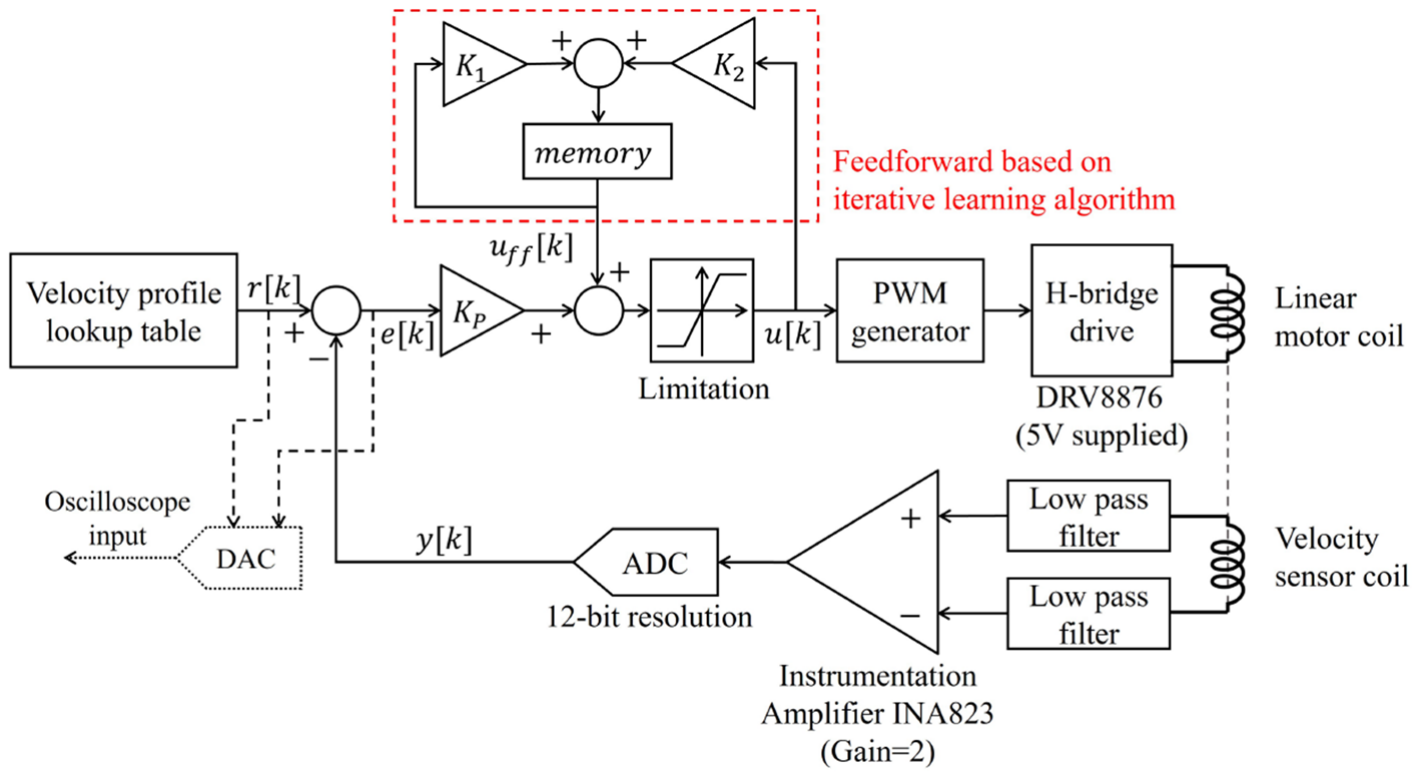
Block diagram of a control system.

**Figure 8:**
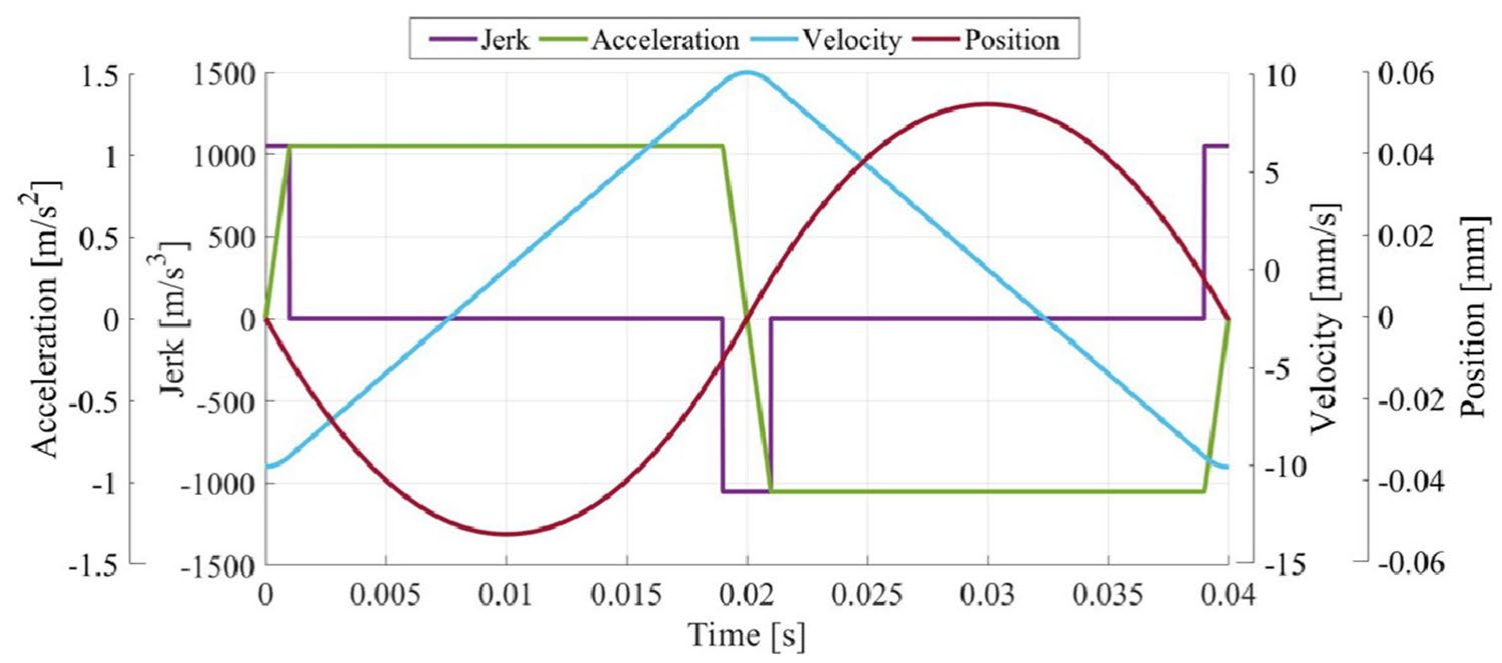
Reference motion profile with jerk limitation.

An overview of the proposed control system is shown in Fig. 7. The LVT signal pair is input into the differential instrumentation amplifier (amplification gain: 2$\times$). Low-pass filters are used to reduce the signal noise of the LVT signal pair before entering the instrumentation amplifier. The output signal of the instrumentation amplifier is then input into the main controller (STM32G431KB, STMicroelectronics), where it is converted into a 12-bit digital value.

The analog-to-digital conversion (ADC) is configured to be triggered and synchronized with a pulse-width modulation (PWM) signal, sampling at the midpoint of the pulse width rather than at the edge of the PWM signal. This approach helps decrease signal contamination caused by switching noise. During each sampling, the ADC processes oversample eight times at a high rate and average these eight samples. Oversampling and averaging are employed to reduce the influence of impulse noise.

An H-bridge switching amplifier (DRV8876, Texas Instruments) was used as the motor driving circuit. The control signal for the switching amplifier is the PWM signal, and the control resolution of the switching amplifier is dependent on the PWM resolution. The PWM signal was generated with 16 times increased resolution by employing the dithering technique, which enabled more precise control of the motor supply voltage. The PWM signal has a high frequency of 85 kHz to minimize the VCM coil current ripple and is configured in complementary mode. This complementary PWM mode enables smoother transitions in the voltage applied to the motor compared to a normal PWM mode.

The control algorithm was based on proportional feedback control. The performance of target tracking was improved by adding a feedforward controller based on an iterative learning algorithm. In the Mössbauer velocity modulator, repetitive motions are performed with a constant velocity profile, and there is not much variation in load. Therefore, an iterative learning method was applied that works well under these conditions^28^, based on calculating the feedforward value that adds the control output of the previous iteration to the new control output. The iterative learning-based feedforward calculation formula is shown in (Eq. 3), and the final control value is calculated using feedforward and feedback control methods in (Eq. 4). All control gains were tuned through trial and error.

3 $$\begin{aligned} u_{ff}[k]&= K_1 \cdot u_{ff}[k-1] + K_2 \cdot u[k-1] \end{aligned}$$

4 $$\begin{aligned} u[k]&= K_p \cdot (r[k]-y[k])+u_{ff}[k] \end{aligned}$$

#### Motion profile design

As mentioned in the “Design requirements” section, the triangular velocity profile is commonly used as the reference of the velocity modulator control. However, the original triangular profile may cause large control errors because the acceleration changes discontinuously near the vertex. Therefore, the basic triangular velocity profile was modified by restricting abrupt acceleration changes (jerk limitation) so that the changes were made to be smooth and continuous (Fig. 8). This enables improved control performance and reduce unwanted vibrations and noises during motion control^29^.

### Experiment

**Figure 9:**
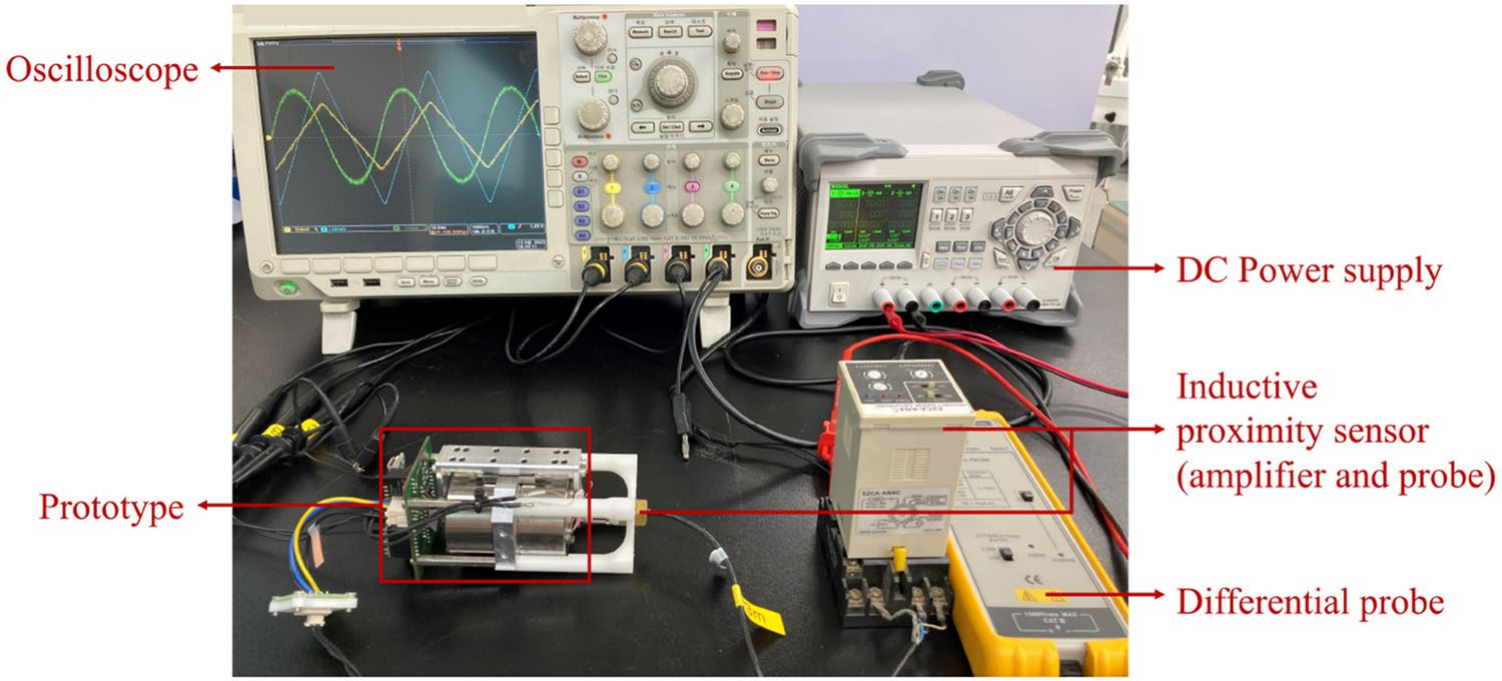
Prototype and experimental setup.

Figure 9 shows the prototype and the experimental setup for evaluating its performance. The specifications of the prototype are summarized in Table 3. The prototype is compact and lightweight compared to commercial products (Table 1). To assess the control performance, the displacement of the moving shaft and dummy source was directly measured using an inductive proximity sensor (E2CA, Omron). This position sensor was employed solely as a verification tool in this study and was not used during the actual operation of the prototype. A differential probe (TA057, Pico Technology) was utilized to monitor the output signal of the position sensor on an oscilloscope (DPO4014B, Tektronix). By differentiating the measured displacement, the actual velocity of the moving shaft can be estimated. The reference signal *r*[*k*] and error signal *e*[*k*] of the controller were converted into analog signals using a digital-to-analog conversion (DAC) and then simultaneously measured on an oscilloscope with the position sensor signal.

**Table 3:** Specification of a prototype.

Parameter	Value	Unit
Moving mass (without dummy)	66.1	g
Total mass (without circuit and dummy)	568.6	g
Mass of a dummy source	4.5	g
Mass of a circuit	25.6	g
Moving stroke (mechanical)	$\pm 1$	mm
Dimension (with dummy)	$53\times 61\times 93$	mm$^3$
Spring constant	1 ($=0.5\times 2 ea$)	N/mm
Power consumption (avg.)	0.55	W

#### Frequency response

**Figure 10:**
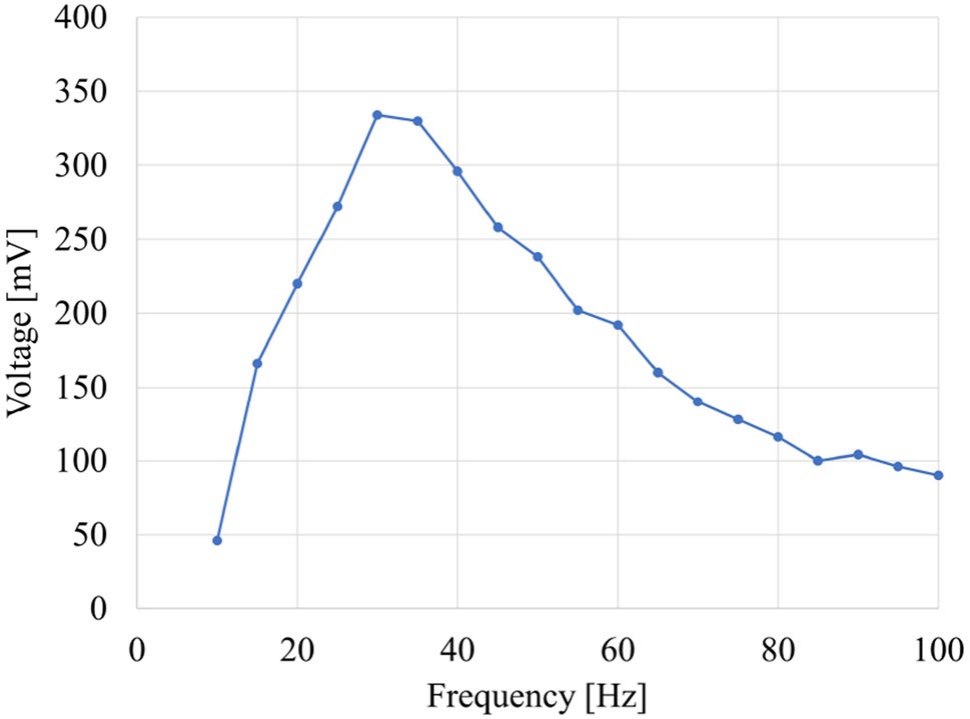
Frequency response of the prototype.

Frequency response (Fig. 10) of the developed prototype (with the dummy source) was measured by stimulating the VCM with a sinusoidal signal with 50 mV of amplitude, provided by a wave generator (EDU33212A, Keysight). The signal induced in the LVT was recorded with the oscilloscope (DPO4014B, Tektronix). As shown in Fig. 10, the resonance is close to 30 Hz (Supplementary Fig. 10).

#### Velocity control performance

To evaluate the sensitivity of the LVT and the control performance of the VCM, profile tracking motion is monitored using a triangular velocity profile with jerk limitation as the target profile, as described in the “Motion profile design” section. To facilitate a direct comparison between the experimental results and the previous EM analysis and simulation results, the amplitude and frequency of the reference profile were configured to match the values used in the EM analysis and simulation (as shown in Fig. 5c,d).

**Figure 11:**
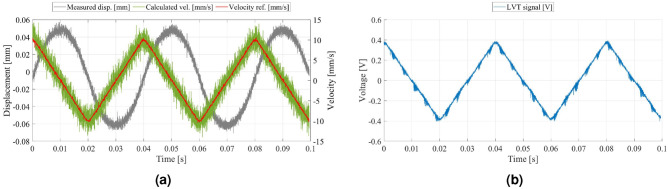
Experimental results. (**a**) Feedback control result. (**b**) LVT signal.

In Fig. 11a, the calculated velocity signal was obtained by differentiating the displacement signal measured from the position sensor. A moving average filter was also applied to reduce high-frequency noise (where the filter conditions include a moving average span: 300 samples; oscilloscope signal sampling period: 10 microseconds; total number of samples: 10,000). When comparing the calculated velocity signal with the velocity reference signal in Fig. 11a, data show that the actual velocity of the moving part closely matches the target profile. When comparing the calculated velocity signal in Fig. 11a with the LVT signal in Fig. 11b, it is observed that the LVT generates a voltage of approximately 0.38 V at 10 mm/s (Supplementary Fig. 11).

## Results and discussion

There is a slight difference between the LVT sensitivity obtained from the experiment (Fig. 11a,b) and the results of the EM analysis and simulation (Fig. 5c,d). This difference may be related to variations in magnetic flux density between them. In the EM analysis, since the geometry of the LVT is assumed to be axisymmetric (toroid magnet with a rectangular section and radial magnetization), the magnetic flux density is calculated to be slightly higher than that of the circular array of bar-shaped magnets (Fig. 3a), resulting in a higher velocity-to-voltage ratio. This is a predictable result and is not a problem that causes degradation of control performance. In fact, the performance of the prototype is not inferior to that of commercial products (Table 1).

**Figure 12:**
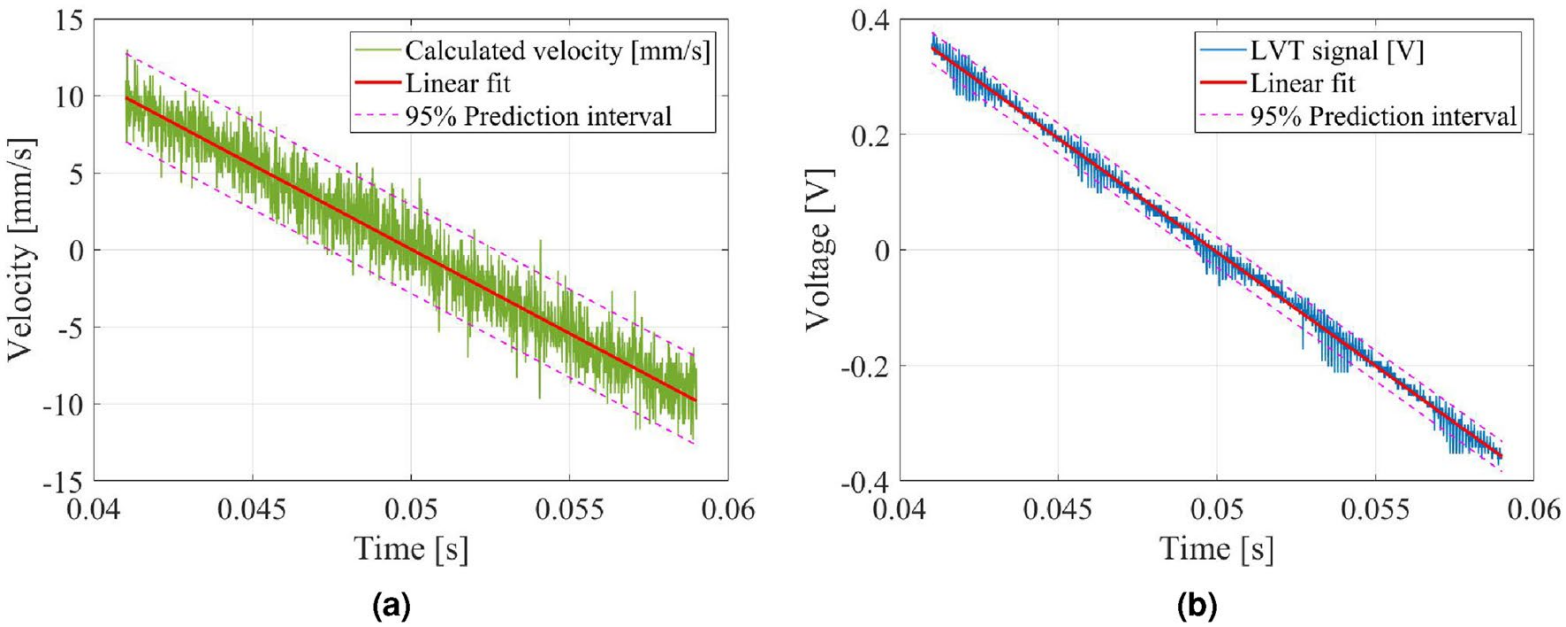
Curve fitting results for a constant acceleration range (0.41–0.59 s). (**a**) Calculated velocity (R$^2=0.940$). (**b**) LVT signal (R$^2=0.996$).

Linearity within the increasing or decreasing sections of the actual velocity signals was assessed through a line fitting, as shown in Fig. 12. This evaluation helps verify the precision and stability of the control over the actual velocity of the moving part while excluding the influence of noise present in the signals. The line fitting process was performed on the two signals; The first was calculated velocity from the displacement signal measured by an inductive proximity sensor; the second is the LVT signal. The data used for fitting were extracted within the constant acceleration range (0.41–0.59 s) of the experimental results.

In the case of the calculated velocity signal, its correlation with a straight line (R-squared) was approximately 0.940 (Fig. 12a). For the LVT signal, the correlation with a straight line was approximately 0.996 (Fig. 12b). These results mean that the developed velocity modulator has good linearity of velocity. In other words, this implies that the velocity control performance of the developed prototype is stable and precise.

When analyzing the causes of errors and noise in both signals, the following conclusions are made. In the former case, the noise is mainly caused by numerical differentiation. In the latter case, the results were affected by the noise induced by the amplifier of the position sensor.

## Conclusion

Here, an advanced velocity modulator was designed and constructed for a portable Mössbauer spectrometer. The developed velocity modulator is more compact and lightweight than previous ones. In addition, it has several distinguishing features as follows:

- Linear bearing with per-loaded springs as linear motion guide and position-limiting mechanism.
- Digital feedback and feedforward control with iterative learning algorithm for improved target tracking performance.
- Switching amplifier with high energy efficiency, which is suitable for portable and long-term operations.

A velocity profile with smooth transitions through jerk restriction was used as a velocity reference. Satisfactory results were reported in terms of target tracking performance and excellent linearity of velocity signals based on the experimental results (i.e., there was a high correlation with the linear fit).

This paper presents a detailed description of how the proposed system was designed by component, which can be used as a valuable reference in the development of velocity modulators for Mössbauer spectrometers. In the future, this prototype will be integrated with a gamma ray detector based on Si-PIN modules and a multichannel analysis system, to fablicate a fully portable Mössbauer spectrometer.

### Supplementary Information

Supplementary Information.

## Data Availability

All data generated or analyzed during this study are included in this article and its supplementary file.
